# A randomized controlled trial of a compassion-centered spiritual health intervention to improve hospital inpatient outcomes

**DOI:** 10.1371/journal.pone.0313602

**Published:** 2025-03-03

**Authors:** Jennifer S. Mascaro, Patricia K. Palmer, Marcia J. Ash, Marianne P. Florian, Deanna M. Kaplan, Roman Palitsky, Steven P. Cole, Maureen Shelton, Charles L. Raison, George H. Grant

**Affiliations:** 1 Department of Family and Preventive Medicine, Emory University School of Medicine, Atlanta, Georgia, United States of America; 2 Department of Spiritual Health, Woodruff Health Sciences Center, Emory University, Atlanta, GeorgiaUnited States of America; 3 Department of Behavioral, Social, and Health Education Sciences, Rollins School of Public Health, Emory University, Atlanta, GeorgiaUnited States of America; 4 Religious Studies, University of South Florida, Tampa, Florida, United States of America; 5 Department of Psychiatry and Behavioral Sciences, Emory University School of Medicine, Atlanta, GeorgiaUnited States of America; 6 Research Design Associates, Inc, Yorktown Heights, New York, United States of America; University Medical Centre Ljubljana (UMCL) / Faculty of Medicine, University Ljubljana (FM,UL), SLOVENIA

## Abstract

**Background:**

Inpatient medical settings lack evidence-based spiritually integrated interventions to address patient care needs within a pluralistic religious landscape. To address this gap, CCSH™ (Compassion-Centered Spiritual Health) was developed to leverage the skillsets of healthcare chaplains to improve patient outcomes through spiritual consultation. Here, we report the results of a randomized, wait-list controlled, pre-registered (NCT03529812) study that evaluated the impact of CCSH on patient-reported depression and explored putative mediators of CCSH’s effects.

**Method:**

Chaplain residents were randomized to be trained in CCSH as part of their clinical pastoral education (CPE) residency in the fall (n =  8) or spring semester (n =  8). After fall training, all residents provided spiritual consultations with hospitalized patients (n =  119; n =  54 seen by CCSH-trained chaplains). Those not yet trained to deliver CCSH provided a traditional consult. Patients’ pre-consult distress was measured using the National Comprehensive Cancer Network Distress Thermometer, and post-consult depression was measured using the Hospital Anxiety and Depression Scale (HADS). Consults were audio-recorded and transcribed verbatim, and we conducted linguistic analyses using LIWC 2015 software to quantify chaplain linguistic behavior.

**Results:**

Patients seen by CCSH-trained chaplains had lower post-consult depression scores (*M* =  4.10, *SD* =  5.04) than patients who were seen by wait-listed chaplains (*M* =  6.12, *SD* =  5.08), after adjusting for pre-consult distress (p = .048). There was also a significant relationship between post-consult depression and chaplain LIWC clout scores (*r* =  -0.24, *p* = .017), a linguistic measure thought to reflect the expressive confidence and other-oriented focus of the speaker. An exploratory mediation model revealed an indirect effect of CCSH on patient depression through chaplain clout language *b* =  -0.11 (90% CI, -.257, -.003).

**Implications:**

These data suggest that CCSH decreases patient depression among inpatients, in part due to CCSH-trained chaplains’ use of more inclusive, confident, and other-oriented language. We connect these findings with current understandings of effective clinical linguistic behavior and reflect on what this work may mean for integrated spiritual health care.

## Introduction

Spiritual health clinicians (SHCs), often referred to as healthcare chaplains, work collaboratively with other healthcare professionals to provide emotional and spiritual support that is culturally sensitive and respectful of diverse religious orientations [1–3]. Extensive research indicates that spiritual consults can improve patient outcomes [4,5], well-being [6], and satisfaction [7–9]. Approximately two-thirds of all hospitals in the United States employ SHCs, who are trained to recognize and respond to the emotional, psychosocial, spiritual, and moral distress of patients and their loved ones, as well as healthcare providers and staff [10,11]. Moreover, SHCs have professional preparation to provide care to people of any or no faith tradition in an increasingly pluralistic and non-sectarian culture [12,13], and they are among the most racially and ethnically diverse members of the interprofessional healthcare team [14]. Together, their preparation, training, and position within the healthcare team make SHCs an ideal group of interventionists to address social, emotional, and spiritual patient care.

Although research demonstrates the value of spiritual consults, the key components that contribute to the effectiveness of chaplaincy spiritual care have yet to be identified [5,15,16]. The increased demands on modern-day SHCs to address broader emotional and social dimensions of care underscore the importance of rigorous research in this area [8]. Correspondingly, spiritual health leaders have prioritized the development and evaluation of evidence-based interventions [17,18]. Most studies to date evaluating healthcare chaplain interventions have identified the skills and activities that chaplains commonly perform and exhibit [ 16,19,20]. For example, one study characterized a taxonomy of common SHC activities, with active listening, providing presence, and demonstrating caring and openness as the most common [21]. Another study used principal component analysis to identify and broadly categorize 59 SHC interventions into religious/spiritual activities and psychological activities, with the former representing interventions unique to chaplains and associated with higher patient satisfaction levels [16].

Although these activities are often referred to as ‘interventions’ in the spiritual health literature, there are very few comprehensive, manualized interventions based on theoretically grounded models of spiritual care. The rigorous development and evaluation of such interventions is vital for optimizing and standardizing spiritual health care, enhancing the education of new SHCs, and testing models of care and change. Motivated by the need for effective manualized approaches to spiritual health care rooted in theory and supported by empirical evidence on the nature and benefit of compassion for patients and the providers who care for them [22,23], we developed CCSH (Compassion-Centered Spiritual Health). CCSH augments spiritual health education using insights and practices of CBCT® (Cognitively-Based Compassion Training), an evidenced-based contemplative training program that draws on the *lojong* tradition of Indo-Tibetan Buddhism and has been shown to increase compassion and decrease depression, loneliness, and the inflammatory response to psychosocial stress [22,24–29]. CBCT combines exercises for stabilizing attention and calming the mind with contemplation of aphorisms, visualizations, self-inquiry, and related meditative exercises for reinforcing and internalizing compassionate perspectives, and it was developed to be accessible to those of any (or no) faith tradition. CCSH prioritizes *compassion* as a core objective of inclusive and integrated spiritual care, and it is designed to strengthen SHCs’ internal reserves of warm-hearted compassion and to skillfully incorporate compassion-based insights and practices for the benefit of patient care-seekers during spiritual health consultations. With respect to clinical spiritual health practice, CCSH is designed to cultivate a SHC’s ability to resist entanglement with suffering, and to augment self-compassion, expanded and inclusive perspective-taking, an accurate and skillful understanding of helplessness, and a balanced need for hope and grief.

Here, we used a randomized, wait-list controlled design that compared patient consultations by CCSH-trained SHCs to consultations by SHCs not yet trained in CCSH. Our primary aim was to evaluate the effectiveness of CCSH in reducing patient depression compared with a standard spiritual health consultation. In addition, we aimed to identify mediating or intermediary variable(s) that account for any observed increased effectiveness of CCSH. For example, if CCSH is effective in improving patient well-being, it may do so by altering the types of language that SHCs use during the consultation, by improving the patient’s perceived experience with the SHC, or both. We used a multi-method approach to examine putative mediators of CCSH effectiveness that included objective measures of chaplain linguistic behavior and patient-reported perceived benefits.

## Methods

### Overview

This evaluation was part of a larger longitudinal, randomized, wait-list controlled study that was pre-registered (NCT03529812), with chaplain-reported depression, anxiety, burnout, and empathy as the primary outcomes (reported in a paper not yet published) of a novel compassion-centered spiritual health training. Here, we examine the impact of CCSH training on patient-reported reductions in depression, pre-registered as a secondary outcome of interest. Because this is the first examination of CCSH and because to our knowledge there are no analogous randomized trials of chaplain interventions delivered to hospitalized patients and compared with standard chaplaincy consult, we did not conduct an *a priori* power analysis. Rather, this was conceived as a convenience sample in which we collected data from as many patients as would consent during chaplain work hours, with a goal of calculating and reporting effect sizes for all observed effects.

The Emory University Institutional Review Board approved the study, and all participants (SHCs and patients) gave written informed consent. SHC residents were randomized to either receive CCSH in the first component of the residency program or to a wait-list group that received CCSH training at the end of the residency program. Importantly, although all 21 SHC residents were randomized, the research was completely voluntary and all SHCs were informed that their participation status would be unknown to their CPE instructors. Most (17) of the 21 residents enrolled in the study, with 8 SHC residents in the CCSH group and 9 SHC residents in the waitlist group enrolling in the study. One SHC resident from the waitlist group withdrew from the study, reporting that they were struggling with the CPE curriculum and with their clinical work and did not feel comfortable being included in the data collection shadows. Thus, the final sample included in the study was n =  8 residents randomized to CCSH and n =  8 residents randomized to waitlist. Importantly, CCSH was built into clinical pastoral education (CPE); thus, the CCSH group did not receive extra training time compared with the wait-list group. While residents randomized to CCSH were receiving their training, residents in the wait-list group completed other CPE didactics supportive of their work as SHCs. Specifically, the wait-list group received instruction in staff support, including varying strategies for reaching out to staff effectively and understanding more about their unique support needs, supplemented by different theories that inform staff support. During this time, both groups continued to receive traditional CPE activities including small group and individualized supervision and reflective case studies.

After CCSH training was complete for the first group, researchers accompanied SHC residents in both conditions during their normal clinical hours as they conducted consultations with hospitalized inpatients. Over the course of data collection, researchers shadowed each SHC resident for at least two hours (average amount of time residents were shadowed was 8.38 hours, SD: 1.33 hours), with shadowing sessions scheduled at random using the following procedure. At the outset of the study, a researcher who was blind to resident group assignment generated a random order of SHC residents. Each week, residents were contacted in that randomized order and scheduled during their scheduled work block during the following week. If a resident was not available that week, they were skipped and contacted again the next week. Shadows proceeded in this way until all residents were shadowed at least once, and then the scheduling was repeated in the same order.

During each shadowing session, researchers accompanied residents during their consultations. Prior to the SHC entering the room for a consultation, a member of the research team obtained informed consent from the patient and then administered a single-item distress measure (described below). If the patient did not want to participate in the study, the research team left the room and the SHC conducted the consult as usual and with no data collection. If the patient consented to be in the research study, the researcher then left the room while the SHC resident conducted a spiritual health consultation with the patient. The consultation was recorded using an audio recorder placed in the SHC’s pocket. After the SHC resident completed the consultation and left the patient’s room, the research team-member administered the post-consult patient-reported outcome measures (described below). Of note, a subset of patients (n =  18) did not complete the patient-reported outcome measures either because they fell asleep during the consultation (in which case researchers did not wake patients), became too drowsy to accurately complete the measures, or they had to be taken for a medical test or procedure (in which case researchers did not delay medical care). Data collection for this component of the study spanned three months, from December 10, 2018, through March 1, 2019, until SHC residents in the wait-list group started CCSH training.

### Setting and participants.

#### Chaplain participants.

SHC residents were full-time trainees enrolled in a year-long CPE program accredited by ACPE: The Standard for Spiritual Care and Education. There were no exclusion criteria for the SHC residents. SHC residents had a mean age of 39.6 (*SD* =  10.2). The social and demographic characteristics of SHC residents (N =  16; 8 CCSH trained, 8 wait-list) are presented in **Table 1**. SHCs conducted a mode of six consultations with patients, ranging from 2 - 11 consultations (*Mdn*: 6; *M*: 6.25, *SD*: 2.28). All consultations were with patients not yet seen by a SHC (i.e., no repeat consults with the same patient were included in this study).

**Table 1:** Social and demographic characteristics of chaplain residents.

		N	%
**Sex**			
	Female	10	63%
	Male	6	38%
**Race**			
	Asian	2	13%
	African American/Black	10	63%
	Afro-Caribbean	1	6%
	White	2	13%
	Other	1	6%
**Relationship**			
	Single	6	38%
	Divorced	3	19%
	Single, living with someone	7	44%

SHC residents in this CPE program were assigned to one of five hospital locations, where they provided consultations with patients of any or no faith as part of their instructional and clinical activities. SHCs conduct consults with patients in three general scenarios. First, they attempt to consult with all inpatients when they are first admitted. Second, SHC consultations occur at the request of the patient or family, and at the request of clinical staff if they are concerned about a patient’s well-being. Third, SHCs also respond to cardiac arrest codes and deaths, and they assist patients in the completion of advance directives and in end-of-life planning. From 2017–2020, staff and resident SHCs in this healthcare system averaged 72,811 patient consultations per year, and they conducted 77,683 consultations with patients in 2019 (the year this study took place).

#### Patient participants.

Patients (n =  119, n =  54 seen by CCSH-trained SHCs, n =  65 seen by non-trained SHCs) were recruited from five acute-care hospitals that are part of one hospital system in a major metropolitan area in the southeastern United States visited by the SHC residents enrolled in our study between August 2018 and March 2019. Patients were eligible for study inclusion if they were at least 18 years of age, English speaking, receiving care on an inpatient unit, and willing to receive a consult from an SHC resident enrolled in our study (unit types and patient demographics can be found in **Table 2**). Patients were excluded if they were determined by the researcher to be cognitively impaired (e.g., unable to answer basic questions), on a ventilator, or were in a room requiring enteric or airborne precautions (e.g., use of an N-95 mask requiring fit testing) to enter (see Supporting Information for consort flow diagram). The clinical, social, and demographic characteristics of patients (N =  101) are presented by SHC training status in **Table 2**. Patients were 56% female and 44% male, with a mean age of 59.3 (*SD*: 17.2; range: 20.6 – 91.7).

**Table 2:** Clinical, social, and demographic characteristics of patients according to whether they received a consult from a CCSH or waitlist SHC.

		CCSH-trained consult		Waitlist consult	
		N	%	N	%
**Sex**					
	Female	25	54%	32	58%
	Male	21	46%	23	42%
**Race**					
	Asian	2	4%	1	2%
	Black	23	50%	24	44%
	White	19	41%	28	51%
	Unknown	2	4%	2	4%
**Relationship**					
	Married	20	43%	25	45%
	Divorced	4	9%	8	15%
	Separated	1	2%	0	0%
	Single	15	33%	18	33%
	Widowed	5	11%	3	5%
	Unknown	1	2%	1	2%
**Admitting Hospital Service**					
	Unknown	6	13%	8	15%
	General Medicine	5	11%	14	25%
	Cardio/Cardiovascular	2	4%	0	0%
	Emergency/ICU	13	28%	21	38%
	Urology/Gynecology	2	4%	2	4%
	Neurology/Neurosurgery	8	17%	3	6%
	Psychiatry	0	0%	4	7%
	Ortho/Rehab Medicine	6	13%	1	2%
	Thoracic/Pulmonary	4	9%	2	4%
**Religion**					
	Unknown	4	9%	5	9%
	Protestant Christian	35	76%	34	62%
	Catholic	1	2%	3	5%
	Muslim	1	2%	0	0%
	Jewish	1	2%	0	0%
	No Preference	4	9%	10	18%
	Non-denominational	0	0%	3	5%

#### Randomization and Blinding.

During the fall unit of CPE, SHC residents were randomized to CCSH or to the wait-list condition. Those in the wait-list group continued to engage with the standard CPE curriculum and received CCSH closer to the end of the CPE residency program. SHC residents were randomized by hospital location using the RANDBETWEEN function in the software Microsoft Excel, such that there were a roughly equal number of SHCs randomized to the CCSH and wait-list groups within each hospital location. All research personnel were blind to chaplain group assignment throughout the entirety of data collection, data entry, and statistical analysis. Importantly, all patients were blind to their SHC resident’s group assignment for the entirety of the consult and were informed during consent: “The purpose of the study is to observe the work that hospital chaplains do and to better understand chaplain-patient interactions.”

#### CCSH (Compassion-Centered Spiritual Health).

CCSH expands spiritual health education, beginning with a 4-week intensive course in CBCT incorporated into CPE. After CBCT training, SHCs learn to deliver the manualized CCSH intervention. The CCSH protocol for a clinical spiritual care consultation is based on a four-stage model. In the first stage, the chaplain prepares for the encounter using a variety of strategies to calm their internal thoughts and feelings and to clarify their intention to offer care and comfort to someone who may be suffering. In the second stage, the chaplain attunes to the relationship with the care-seeker (a patient or their loved one, a provider or staff member) by attending to the person’s emotional state as well as to the factors that might contribute to or alleviate their distress. Observations at this stage guide decisions about how to proceed through the intervention, and the SHC may return to this stage of CCSH whenever they sense changes in the care-seeker’s level of distress that should inform decisions about how to intervene as a care-responder. The third stage of CCSH involves accessing compassion, and this stage equips the chaplain to offer practices, perspectives, and insights for facilitating compassion, warm connection, and emotional resilience. The concluding stage of CCSH is entrusting the care-seeker, which draws the CCSH consultation to a close while acknowledging the self-efficacy and aspiration of the care-seeker to maintain their own sense of hope, resilience, resolve, and compassion, and by recalling any resources and supportive circumstances that were identified during the consult.

### Measures.

#### Patient self-report measures.

The National Comprehensive Cancer Network Distress Thermometer [30] was administered prior to each chaplain consultation. The distress thermometer is a single-item distress screening tool that asks patients to indicate how much distress they have been experiencing in the past week, including that day, using a scale from 0 (“No distress”) to 10 (“Extreme distress”). It compares favorably with longer measures used to screen for distress [30]. Although developed for use in oncology contexts, the Distress Thermometer has been used with and validated in other patient populations [31–33].

Upon completion of the chaplain consultation, we administered the patient experience subscale of the Scottish Patient Reported Outcome Measure (PROM) [34,35] as a measure of patient-reported experience with the chaplain consultation. Patients reflect upon and rate their experience with the chaplain using a five-point (0 =  “None of the time”, 4 =  “All of the time”) scale and four-items (e.g., “During my time with the chaplain I felt my situation was understood and acknowledged.”). Cronbach’s α indicated acceptable internal reliability, α = .77.

We also administered the Hospital Anxiety and Depression Scale (HADS) [36–38], a 14-item questionnaire that is widely used for detecting anxiety and depressive disorders in a hospital setting. Participants use a four-point (0 =  “Not at all”, 3 =  “Nearly all the time”) response category to rate how they have been feeling in the past week, such that possible scores range from 0 to 21 for depression (e.g., “I feel as if I am slowed down”). Cronbach’s α indicated high internal reliability, α = .81.

#### Audio recordings.

For each chaplain consultation, the audio recording was transcribed and then independently checked by two researchers. Discrepancies in transcription were resolved by the research team as needed. Eighteen additional consults were transcribed, but post-consult patient self-report data were missing (reasons described above) and thus are not included in the analysis (n =  101, n =  46 seen by CCSH-trained SHCs, n =  55 seen by non-trained SHCs). Here, we focused on only chaplain speech (findings related to patient language are reported elsewhere).

#### Linguistic analysis.

To analyze the language recorded during the chaplain consultations, we used the LIWC 2015 software [39], a widely used and extensively validated word-count based text analysis tool in the social sciences. LIWC matches words from the text with entries in an internal dictionary. Dictionary items are categorized into a set of grammatical and semantic domains. LIWC then calculates the percentage of total words from the text that sort into each domain, such as affect words (e.g., sadness), social words (e.g., family), core drives and needs (e.g., achievement), and biological processes (e.g., body). The variables based on these analyses are expressed as the percentages of all words spoken by the chaplain in that consultation. For example, if there are 25 “body” words in a sample of 1,000 words, the LIWC output for body-associated speech would be 0.025 or 2.5%.

In addition to the relative frequency of domain-specific words, LIWC scores each text file along four summary variables: analytical thinking, clout, authenticity, and emotional tone. Scoring for each of the summary variables is based on previously published findings in which several domains are aggregated and converted to a standardized percentile score for that summary variable that can range from 0 to 100 [39]. Analytic thinking is thought to reflect the degree of formal or logical thinking on the part of the speaker, and it is calculated based on the formula: [articles +  prepositions - pronouns - auxiliary verbs - adverb - conjunctions - negations] [40]. Clout is thought to reflect the expressive confidence of the speaker and it is calculated based on the formula: [we-words +  you-words +  social words - i-words - swear words - negations - differentiation words] [40]. Authenticity is a measure of the degree to which someone is personal, vulnerable, or humble in their speaking. Emotional tone is a measure of the relative ratio of positive to negative emotion words, such that the higher the number, the more positive the tone. For this study, we focused on the four summary variables to reduce problems related to multiple comparisons, to reduce the number of observable variables in our formative mediation analyses, and because these variables aggregate the linguistic categories of greatest interest to our study, including pronoun use, affective language, and the use of social words.

#### Data analysis.

The data were analyzed using Statistical Package for the Social Sciences (SPSS) software, version 28.0. We used expectation maximization [41] to estimate missing items (missing items never accounted for more than 5% of total data) using other items within the scale as predictor variables. Assumption of distribution normality was assessed with the Shapiro–Wilk test. With a statistically significant Shapiro-Wilk test, bootstrap simulation using 500 samples was used to assess normality of the underlying sampling distribution of the measure to determine if use of parametric statistical tests based on the Central Limit Theorem would be appropriate. Standardized residuals for the patient depression measure were generated to control for pre-consultation levels of distress. We did this using linear regression, with depression as the dependent variable and scores on the pre-consultation distress thermometer as the independent variable.

We conducted linear mixed model analyses to evaluate the effect of chaplain training status (CCSH vs. wait-list) on patient-reported depression after the consultation, controlling for pre-consult distress. Maximum likelihood was used to estimate the model. To assess the extent of non-independence of chaplain observations given that chaplains each conducted multiple consultations, intraclass correlation coefficient (ICC) and design effect were calculated. With a small ICC = .09 and average cluster size =  7.12, the design effect =  1.59. It is suggested that with low ICC and design effect less than 2, multi-level model analysis is not needed [42]. However, to be cautious, we included chaplain as a first-level predictor to account for possible non-independence of chaplain observations.

Next, we conducted exploratory mediation analyses using the PROCESS macro for SPSS. [43] First, we conducted Pearson product-moment correlations between all study measures to identify putative mediators, which included LIWC summary variables (analytic, clout, authenticity, and tone), patient-reported experience with the chaplain consultation, and patient depression residuals. An initial regression explored the effects of chaplain group on patient post-consult depression. Next, we fit a mediation model: Path *a* explored the effects of chaplain group on chaplain linguistic behavior. Path *b* explored the effect of chaplain linguistic behavior on patient depression, controlling for chaplain treatment group. The indirect effect of chaplain group on patient depression with chaplain linguistic behavior as mediator is the product of the *a* and *b* coefficients. Tests of significance were two-sided, and results with *p* < .05 were considered statistically significant. Bias-corrected bootstrapping was used to estimate the confidence intervals for the mediating effects of putative mediators on patient depression residuals [44]. Although sample sizes required to detect mediation effects with bias-corrected bootstraps are smaller than most other tests, our sample size is most likely underpowered, thus 95% and 90% confidence intervals are reported for indirect effects [45].

## Results

### Patient-reported outcomes

Prior to SHC consultation, patients reported distress ranging from 0 to 10 (*M* =  5.94, *SD* =  3.25), with a modal score of 10 and 74% (n =  75) reporting a distress score of 4 or higher, the cutoff commonly used as an indicator of clinically significant distress [46]. After the SHC consultation, patients reported depression scores ranging from 0 to 20 (*M* =  5.20, *SD* =  4.25) and anxiety scores ranging from 0 to 18 (*M* =  6.77, *SD* =  5.02).

### Effects of chaplain training status on patient depression

There was a statistically significant effect of chaplain training on patient depression, *b* =  2.03, (95% CI, 0.02, 4.03), *p* = .048 *d* =  0.40. Patients seen by CCSH-trained chaplains had lower post-consult depression scores (*M* =  4.10, *SD* =  5.04) than patients who were seen by wait-listed chaplains (*M* =  6.12, *SD* =  5.08). Means and standard deviations were adjusted by pre-consultation distress scores.

#### Association between chaplain language, patient depression, and patient satisfaction.

Bivariate correlations explored whether patient depression residuals were associated with putative mediators of chaplain training (**Table 3**). These analyses revealed significant relationship between post-consult depression and both chaplain clout language (*r* =  -0.24, [95% CI, -0.41, -0.04], *p* = .017) and chaplain emotional tone (*r* =  -.35, [95% CI, -0.51, -0.16], *p* < .001). Post-consult depression was not significantly associated with patient-reported experiences with the chaplain.

**Table 3:** Bivariate correlations between pre-consult patient distress, post-consult patient depression and depression residuals (accounting for pre-consult distress), and hypothesized mediators of CCSH effects.

	Pre-consult distress	Post-consult depression	Std. resid. post-consult depression	Patient experience	LIWC analytic	LIWC clout	LIWC authentic	LIWC tone
Pre-consult distress		**.295** **	0.000	-0.065	0.022	-0.011	-0.013	-0.194
Post-consult depression	**.295** **		**.955** **	-0.093	0.129	**-.230** *	-0.027	**-.387** **
Std. resid. post-consult depression	0.000	**.955** **		-0.077	0.128	**-.238** *	-0.024	**-.345** **
Patient experience	-0.065	-0.093	-0.077		0.105	-0.002	-0.167	-0.145

**. Correlation is significant at the 0.01 level (2-tailed).

*. Correlation is significant at the 0.05 level (2-tailed).

#### Mediators of chaplain training and patient depression.

**Fig 1:**
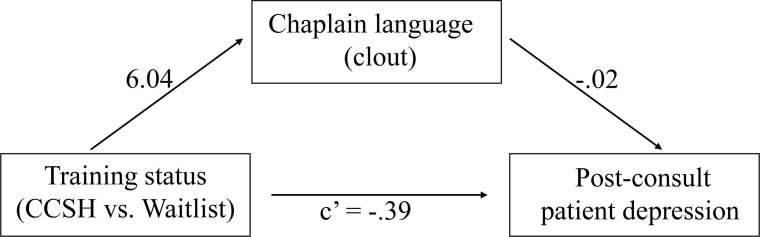
Path diagram depicting the mediation model with clout language as a mediator of chaplain training on patient depression after the consult.

In initial regressions, CCSH training was associated with lower patient depression scores (*b* =  -0.50, [95% CI, -0.88, -0.12], *p* = .011) and clout language was associated with lower patient depression scores (*b* =  -0.03, [95% CI, -0.05, -0.01], *p* = .017). In the mediation model (**Figure 1**), CCSH training positively influenced clout language (*b* =  6.04, [95% CI, 2.56, 9.51], *p* < .001) (Path *a*). When controlling for CCSH status, clout language had a reduced effect on patient depression (*b* =  -0.02, [95% CI, -0.04, 0.003], *p* = .091) (Path *b*). When controlling for clout language, CCSH had a reduced effect on patient depression, (*b* =  -0.39, [95% CI, -0.78, 0.02], *p* = .060, *d* =  39). The indirect effect of CCSH on patient depression through chaplain clout language *b* =  -0.11 [95% CI, -0.31, 0.04; 90% CI, -0.26, -0.003] was statistically different from zero at the 90% level.

## Discussion

Within modern health systems, spiritual health care and healthcare chaplaincy increasingly require rigorous research designs and evaluation to demonstrate evidence-based effectiveness and value [17,18,47]. Here, we examined the effectiveness of CCSH, a newly developed program that augments spiritual health education and best practices with evidence-based compassion training practices and insights. In planned exploratory analyses, we also examined putative mediators of the effect of CCSH on patient depression.

The primary finding of our study is that, after adjusting for patients’ pre-consultation distress scores, patients seen by CCSH-trained SHCs had lower depression scores. Interestingly, the depression measure used here (HADS) asks patients about the extent to which they have felt depression symptoms “in the last week.” Because the chaplain consult cannot change the way a patient felt in the 6 days prior to the consult occurring, one interpretation of this finding is that an effective chaplain consult has an effect via altering a patient’s “mood congruent recall bias” — that is, the phenomena by which perceptions of how we felt in the past are influenced by how we feel right now [48,49]. The finding suggests that consultations with a compassion-trained chaplain led patients to interpret their mood and state of mind more positively for the preceding week. Although this study did not assess momentary mood/affect, this finding may suggest that encounters with CCSH-trained chaplains were more successful at altering momentary affect.

Our interpretation of this finding is that while standard SHC consults were effective, SHCs trained in CCSH provided greater benefit to patients in terms of reducing depression symptoms. CCSH is a comprehensive program incorporated into healthcare chaplain education that includes skills and practices to augment emotional awareness and emotion regulation, to improve attunement to the dyadic and dynamic relationship with the care-seeker, and to intentionally cultivate warm-hearted compassion. With our research design, we cannot definitively identify which of the active training ingredient(s) of CCSH account for the increased SHC effectiveness. We surmise that the effects of CCSH are related at least in part to the intentional and systematic cultivation of resilient warm-hearted compassion toward care-seekers in a clinical context for three reasons based on previous research. First, in a previous mixed-method study of a subset of the SHC residents included in this study, residents reported that CBCT led to profound insights, changes, and change processes related to their experience of compassion [50]. Second, in a previous study conducted with parents of young children, children of parents randomized to take CBCT had reduced cortisol compared to children whose parents were randomized to a wait-list group [51]. This finding that CBCT benefits those downstream of the practitioners themselves is consistent with our finding here, and it is possible in both studies that care-seekers’ (young children and patients) well-being was bolstered by interacting with care-responders (parents and SHCs) who had increased resilience and compassion. Third, in a previous study we found that SHC clout language, the linguistic behavior associated with CCSH training in the current study, was associated with SHC’s self-reported compassion [52]. An extensive and growing body of research highlights the importance of caregiver compassion for patient well-being and clinical outcomes [53,54], and our findings are consistent with this in as much as the systematic cultivation of resilient compassion in CCSH appears to improve SHC effectiveness. However, it is likely that other aspects of CCSH are also important. For example, in our mixed-method study, residents also reported that CBCT increased their self-compassion and improved their ability to identify and understand their emotions [50], both of which may be of benefit in their consults with patients. Future narrative and ethnographic research will be important to examine the multi-faceted training effects of CCSH coupled with CPE and to understand the active ingredients of using CCSH with patients.

Here, we focused on patient depression after the consultation given its impact on patient well-being and long-term clinical prognosis [55–58]. Moreover, depression symptoms are highly relevant to spiritual health consultations. The largest study to date that examined chaplain consults found that anxiety, sadness, and grief were the most common reason for chaplain referral [1,2]. Although research in spiritual health care has increased in breadth, depth, and rigor, the field of spiritual health care has historically focused primarily on process outcomes (e.g., meaning-making) rather than on patient outcomes related to clinical effectiveness [47]. By evaluating depression symptoms, our study demonstrates that attention to and optimization of SHC training has downstream impact on clinical outcomes of high importance to hospital medicine.

In addition to being primarily process-oriented, researchers and practitioners in the field of spiritual health and chaplaincy research have also expressed ambivalence about the need for manualized intervention and problem-focused care [47]. Concerns over this approach to chaplaincy include the objection that more medicalized, outcome-oriented spiritual care may lead to dehumanization of the care-seeker [59] or to a reductionistic, one-size-fits-all view of the patient that glosses over patient uniqueness and limits the SHC’s flexibility [47]. For this reason, some in the field have rejected manualized and evidence-based interventions such as CCSH. In fact, often the term ‘intervention’ is used to describe skills, activities, or behaviors carried out by the chaplain rather than a standardized, theory-based system of treatment. For example, one study identified 59 spiritual health interventions, which fell broadly into two independent groups: interventions involving a religious/spiritual dimension (e.g., prayer, facilitated life review), and those involving a psychosocial dimension (e.g., cultivated trust, advocacy) [16].

Importantly, CCSH was designed to have the flexibility to contain any combination of these skills/activities, yet it is a manualized, integrated, and theoretically grounded intervention. In this way, CCSH is analogous to a handful of other manualized chaplain-led interventions [60,61]. The chaplain-led manualized intervention that has the most established evidence base is the Spiritual Assessment and Intervention Model (AIM), which emphasizes assessment and the relational interaction between SHC and care seeker built on the patient’s fundamental needs for meaning and direction, self-worth and belonging, and love [60,62]. We view CCSH as containing an intentional cultivation of compassion and compassion-based resources (both for the SHC and the care seeker) as well as contemplative practices and insights to augment emotion regulation and interpersonal attunement. Importantly, both CCSH and Spiritual AIM have intentionally structured flexibility to respond to the idiosyncratic concerns of patients within a pluralistic landscape. This characteristic of manualized interventions will be crucial to mitigate concerns over what some characterize as a more medicalized approach to chaplaincy.

In our formative analysis of putative mediators, the model suggested that an indirect effect at a 95% confidence was not detectable with the sample size we had. However, at a 90% confidence interval, we detected an indirect effect of chaplain clout language as a mediator of the relationship between training status and patient depression. Given the magnitude of the effect size of chaplain group status on patient depression and of chaplain language on patient depression, the study was likely underpowered to detect significance at the 95% level [45]. Moreover, the magnitude of the training status effect on patient depression was very large, and thus controlling for this effect likely prevented detection of a significant mediation effect at the .05 level. Larger studies will be important to definitively test the process model indicated by this study, and we hypothesize that chaplain clout will emerge as an important mediating variable for studies of CCSH and for chaplaincy intervention more generally.

This work also contributes to what is known about chaplaincy process and about the link between process and outcome. Here, we use novel methods to link patient outcomes with relatively objective aspects of the SHC process, an approach recently championed in both chaplaincy and psychotherapy research [47,63]. We found that SHC clout scores were associated with training status and indirectly accounted for the increased benefit of CCSH training on patient depression. The LIWC clout formula was developed based on a body of research that identified linguistic behavior associated with social status and reflecting expressive confidence, inclusivity, and other-centeredness [64]. Specifically, people of higher status tend to use more social words (e.g., *everybody*, *family*) and fewer negation words (e.g., *never*, *shouldn’t*). They also tend to exhibit a different pattern of pronoun-use: fewer first-person singular [e.g., *I*, *me*] and impersonal pronouns [e.g., *it*]; more first-person plural [e.g., *we*] and second-person singular pronouns [e.g., *you*] [64].

Importantly, the clout metric is not thought to tap into a *desire* or *grasping* for clout, status, or power. Instead, the clout score is thought to reflect the linguistic behavior exhibited by people who demonstrate relatively high levels of leadership, confidence, and status during interpersonal interactions. In our research with an overlapping group of SHCs, clout scores were associated with SHC’s compassion capacity, a latent construct composed of self-reported compassion satisfaction, burnout, and empathy [44]. Spiritual care involves transcendence of one’s own religious, sociodemographic, and experiential perspective, which is notoriously difficult – perhaps impossible – to operationalize and quantify. We do not believe the effectiveness of an SHC can be reduced to their linguistic behavior. However, our findings point to clout as a *linguistic footprint* of clinical confidence in orienting towards patients that may indicate an effective spiritual health consult and be trainable through manualized interventions such as CCSH. Surprisingly, patient-reported experience with the SHC did not mediate the effect of CCSH on patient outcomes, further highlighting the importance of relatively objective measures such as linguistic behavior for understanding the process of effective spiritual health consulting.

### Limitations.

There are several important dimensions of spiritual care that were not captured by the present study. For example, nonverbal communication, broad dyadic and relational dynamics, meaning-making, and unique progressions or processes of change are vital to spiritual care interactions; however, these components exceeded the scope of the present study. Related, we did not conduct a qualitative analysis or examine the context or themes associated with the clout linguistic category, which makes definitive interpretation challenging. Research is underway to build upon these findings by examining the context and thematic importance of clout language within spiritual health consultations. Research is also underway to evaluate the impact of CCSH on SHC residents, themselves, an important line of research to definitively test the possibility that patient benefits of CCSH are derived from benefits observed in the SHC residents. Another limitation of this study is that we did not evaluate SHCs’ fidelity to CCSH. A psychometrically sound fidelity coding manual for CCSH is newly available, and future work investigating the effectiveness of CCSH will benefit from evaluating whether intervention fidelity is associated with outcomes. We did not assess SHCs’ religious affiliations or SHC-patient religious concordance, nor do we have the statistical power to examine whether SHC demographics or SHC-patient religious concordance moderates the effects of group status. Previous research suggests that faith/religious concordance between SHCs and patients does not affect patient satisfaction [65], minimizing the concern over this nevertheless important factor. Moreover, we did not assess SHCs’ linguistic backgrounds, and while some of the SHCs were multi-lingual, the study was limited to SHC consultations conducted in English. These data were collected from 16 SHC residents providing spiritual consultations in one hospital system. It is not clear that these findings would be generalizable to spiritual consultations conducted in other regions or cultures. Nevertheless, the volume of clinical interactions with patients each year makes understanding the effects and mediators of chaplain effectiveness, even in this restricted context, important. Additionally, the research was limited to the SHC residents who consented to be in the study. Although most (16 of 21) residents – and equal numbers from the CCSH and waitlist groups – enrolled in the study, it is possible that SHCs who did not participate in the research were different than those whose did not enroll.

A final limitation is related to our prioritizing patient participants’ well-being and access to timely medical care, especially given that many patients enrolled in the study were seriously ill, hospitalized for serious and/or multifarious concerns, and receiving complex and multi-modal treatments. In aiming for brevity of assessment prior to SHC consultation, we opted to use a one-item distress thermometer to adjust for pre-consult distress/depression, which was different from the HADS measure of depression used after chaplain consultations to measure the effectiveness of the intervention. We chose to only administer the single-item state measure of distress prior to the consult for ethical reasons, as many patients in the study were highly distressed and seeking immediate SHC intervention. Previous studies have found that the Distress Thermometer is highly correlated with the depression subscale of the HADS [31,66,67], indicating that controlling for pre-consult distress with the Distress Thermometer helps account for pre-consult symptomology.

### Conclusion.

This mixed-method study used a rigorous study design and validated clinical measures to evaluate CCSH in the hospital setting. Our findings indicate that CCSH is an effective model of spiritual care for reducing inpatient depression symptoms, and the data is suggestive of an indirect effect of CCSH-trained chaplains’ greater use of confident, other-oriented, and social language during the consultation. Larger studies in diverse hospital systems are important to replicate and extend these findings, and this study design can be used to generate new knowledge about spiritual care and to optimize SHC training and practice.

## Supporting information

S1 Text CBCT Chaplains protocol. (DOCX)

S2 Text CONSORT-2010-Checklist_Mascaro. (DOCX)
