# Supplementary material for: A randomized controlled trial of a compassion-centered spiritual health intervention to improve hospital inpatient outcomes
Source: PLoS One. 2025 Mar 3;20(3):e0313602. doi: 10.1371/journal.pone.0313602 (PMC11875371; doi:10.1371/journal.pone.0313602)
Supplement: S1 Text — (DOCX) [file pone.0313602.s001.docx]

Exploring the Effect of Cognitively-Based Compassion Training (CBCT) on the

Empathic Accuracy and Resilience of Spiritual Health Clinicians

**Investigators:**

Geshe Lobsang Negi, PhD

Charles Raison, MD

George Grant, PhD

Jennifer Mascaro, PhD

Kim Palmer, BCC

Marianne Florian, MA, MTS

**Principal Investigator:**

Jenny Mascaro, Ph.D.

Atlanta, GA 30322

Phone: 404-558-4461

Fax: 404-727-2860

Email: [jmascar@emory.edu](mailto:jmascar@emory.edu)

**Principal Contact:**

Jennifer Mascaro

**Short Title:** CBCT - Chaplains

**Draft Date:** June 22, 2018

**Synopsis:**

Based on a wealth of research demonstrating the associations between physical health and psychosocial well-being, modern health care in the United States is characterized by an increasingly patient-centered model of care that places a premium on the holistic treatment of the patient as a physical, psychosocial, and spiritual whole. Hospital chaplains play a vital role in delivering emotional and spiritual care to a broad range of both religious and non-religious patients for a wide variety of stressors, and extensive research indicates that spiritual consults impact patient outcomes and satisfaction. However, there is remarkably little research on the “active ingredients” of chaplaincy spiritual care, and a subsequent lack of standardization and best-practice guidelines informing chaplain training and chaplain spiritual consulting. CBCT ® (Cognitively-Based Compassion Training) is a secularized compassion meditation program adapted from the Tibetan Buddhist mind training (lojong) tradition, and it may be an ideal addendum to both chaplain training programs and to the spiritual care consults provided by Emory University hospital chaplains to approximately 100,000 patients each year. Here we propose a pilot study to explore the feasibility and impact of incorporating CBCT into the educational curriculum for chaplain residents. To this end, we will (1) examine the impact of CBCT on burnout, compassion, and empathic accuracy among chaplains in training, (2) explore whether the impact persists through the end of the resident training, (3) evaluate the timing of the training within the existing curriculum to maximize its impact, (4) evaluate whether CBCT-informed interventions improve patient outcomes, and (5) explore chaplain and patient mediators of the impact of spiritual care consults on patient outcomes. Data acquired in this pilot study will be used to estimate efficacy and to inform a randomized controlled trial.

1. **Background**


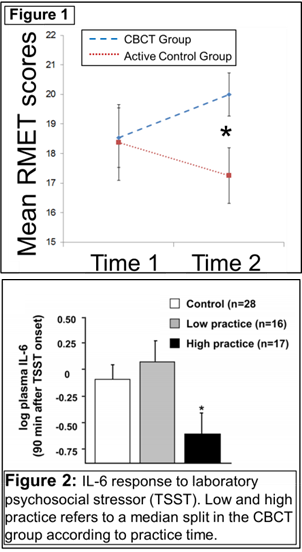
Previous research indicates that CBCT (Cognitively-Based Compassion Training) improves empathic accuracy[^1^](#_ENREF_1) (Figure 1), enhances well-being[^2^](#_ENREF_2)^,^[^3^](#_ENREF_3), and attenuates the pro-inflammatory response to psychosocial stress[^4^](#_ENREF_4)^,^[^5^](#_ENREF_5) (Figure 2). CBCT is a secularized compassion meditation program adapted from the Tibetan Buddhist mind training (*lojong)* tradition, and it may be an ideal addendum to the spiritual caregiving provided by Emory University hospital chaplain residents to approximately thousands of patients each year.

Chaplains are exposed to a great deal of suffering when they provide spiritual care to hospitalized patients and their families. Chaplain residents, who are typically in their first full year of exposure to hospital chaplaincy, can be particularly impacted by the suffering to which they bear witness[^6^](#_ENREF_6). CBCT is anticipated to improve empathic accuracy and resilience in the face of suffering among chaplain residents. The compassion component of CBCT will help the chaplain develop and practice skills of compassion while the cognitive component of CBCT enhances emotional regulation and allows the chaplain to avoid becoming overwhelmed by empathic connection in the presence of another’s suffering. This allows for a compassionate response in which the chaplain is able to “hover in a sweet spot, empathic but not over-aroused.”[^7^](#_ENREF_7) If CBCT is shown to provide long-lasting benefit to chaplain residents, then the chaplaincy field as a whole will have a powerful new tool for the health and retention of healthcare chaplains.

The Emory University Spiritual Health department will incorporate CBCT into their training curriculum using a phased approach beginning in Fall 2017, which provides the opportunity to compare students receiving the CBCT addendum with those receiving traditional chaplaincy training. Here we propose a naturalistic study that examines the impact of incorporating CBCT into Emory’s spiritual caregiver training program. To this end, we will examine the effect of CBCT on empathic accuracy and resilience by comparing the first group of students who receive CBCT-informed training with chaplains who receive standard training in spiritual caregiving and receive CBCT later in the residency year. Data on empathic accuracy will be collected using an existing validated task that measures the degree of correspondence between chaplain-reported emotional states of individuals relaying illness-related experience on video and the emotional states reported by the individuals themselves at the time of video recording[^8^](#_ENREF_8). Data on chaplain resilience will be collected using self-report questionnaires measuring compassion fatigue, compassion satisfaction, empathy, anxiety, and depression.

**a. Specific Aims**

**Specific Aim 1: To estimate the effect of receiving CBCT on empathic accuracy and resilience among chaplain residents providing spiritual care to hospitalized patients.**

We will conduct a naturalistic study of up to 30 chaplain residents serving the Spiritual Health department of Emory Healthcare, and who will receive CBCT during the first unit of their year-long residency (the early-start group) or approximately midway through the residency year (the delayed-start group). Empathic accuracy and resilience will be measured for all participants before the early-start group receives CBCT (baseline, or T0); after the early-start group completes CBCT (T1); before the delayed-start group receives CBCT (T2), and after the delayed-start group completes CBCT (T3). See **Figure 3** for study schematic. The change in mean score of empathic accuracy and resilience measures from T0 to T1 among participants in the early-start group will be compared to the change in mean score among participants in the delayed-start group over the same time period.

**Specific Aim 2: To evaluate whether the effect of CBCT among chaplain residents persists over the residency year.**

For participants in the early-start group only, the change in mean score of empathic accuracy and resilience measures from T0 to T1 will be compared to the change in scores over the T1 to T2 and T2 to T3 time periods..

**Specific Aim 3: To evaluate whether the effects of CBCT depend on the timing of program delivery.**

The change in mean score on empathic accuracy and resilience measures from before to after receipt of the CBCT program among participants in the early-start group (from T0 to T1) will be compared to the change in mean score on empathic accuracy and resilience measures from before to after receipt of the CBCT program among participants in the delayed-start group (from T2 to T3)**.**

**Specific Aim 4: To evaluate whether CBCT-informed interventions improve patient outcomes.**

Upon completion of their training program, chaplain residents will provide spiritual consults to patients throughout the Emory hospital system. We will conduct comprehensive assessments in order to examine whether patient (n = 300) outcomes are improved for those receiving chaplain spiritual care augmented by CBCT, in comparison to those receiving spiritual care from chaplains who receive CBCT later in their residency. To this end, study personnel will shadow chaplain residents in both groups and will administer self-report assessments of negative symptomology (depression, anxiety, and loneliness) and well-being (quality of life, hope) to consented patients. In addition, study personnel will audio record chaplain-patient interactions. Patient outcomes will be gathered from retrospective chart reviews and will include use of opioid pain medication, duration of stay, patient’s denial of care, and patients’ Press Ganey satisfaction scores.

**Specific Aim 5: To explore chaplain and patient mediators of the impact of spiritual care consults on patient outcomes.**

To examine mechanisms that mediate improved patient outcomes following compassion-informed spiritual consults, chaplains will wear an ambulatory assessment device that records chaplain-patient interactions. Recordings will be coded for (1) use of, and adherence to, the CBCT-informed modules, and (2) compassion language and action. Mediation models will test whether chaplain characteristics, patient characteristics, or chaplain-patient interactions account for improved patient outcomes.

**2. Design**

1. **Sample:**

Emory Healthcare chaplain residents will be asked to view the empathic accuracy video scenarios and complete questionnaires at four time points during the residency year. Chaplains will be asked to wear ambulatory recording devices that record chaplain-patient interactions.

Emory Healthcare patients will be asked to complete self-report assessments before and after their Spiritual Health consult. Patients will be informed of chaplains use of ambulatory recording device for research purposes.

No children, prisoners or other vulnerable populations will be included in this study.

*Inclusion Criteria*: Chaplain residents with Spiritual Health at Emory Healthcare or patient of Emory Healthcare.

*Exclusion Criteria*: None

b. **Setting:**

Consenting chaplains will view the empathic accuracy videos and complete the study questionnaires at one or more of the Emory Healthcare campuses (EUH, EUHM, Saint Joseph’s, Emory Johns Creek, and Wesley Woods).

Consenting patients will complete self-report assesments and spiritual health consults at one or more of the Emory Healthcare campuses (EUH, EUHM, Saint Joseph’s, Emory Johns Creek, and Wesley Woods).

c. **Recruitment:**

Chaplain residents and Emory Healthcare patients will be approached in person at the Emory Healthcare campuses by a researcher and will provide written consent.

d. **Procedures**:

Participants will be approached by a researcher who will explain the study to them and have them sign an informed consent document if they understand the study component and are interested in participating. Chaplain resident participants will receive the CBCT as part of their clinical pastoral education (CPE) curriculum. Chaplain resident participants will receive the CBCT either in the first educational unit (the early-start group) or approximately midway through the residency year (the delayed-start group). Residents in the early-start group will meet virtually for a didactic approximately every two weeks to discuss their residency experience in the context of their CBCT knowledge and caregiving approach. Residents in the delayed-start group will meet for a similarly structured and scheduled didactic on spiritual health topic(s) unrelated to patient care. After the delayed-start group receives CBCT approximately midway through the residency year, the students in the delayed-start group will also meet virtually for the CBCT-focused didactic to discuss their residency experience in the CBCT context and the early-start students will receive the spiritual health didactics on topic(s) unrelated to patient care. All didactics will be 1.5 hours in length. All chaplain resident participants will view the empathic accuracy video and complete the questionnaires at four times during the residency year: before the early-start group receives CBCT (baseline, or T0); after the early-start group completes CBCT (T1); before the delayed-start group receives CBCT (T2), and after the delayed-start group completes CBCT (T3). A subset of the chaplain residents will be interviewed at three time points relative to their training in CBCT. The interviews consist of open-ended questions to further query their learning experiences with CBCT. Researchers will compare differences in the mean scores of empathic accuracy and resiliency measures over time.


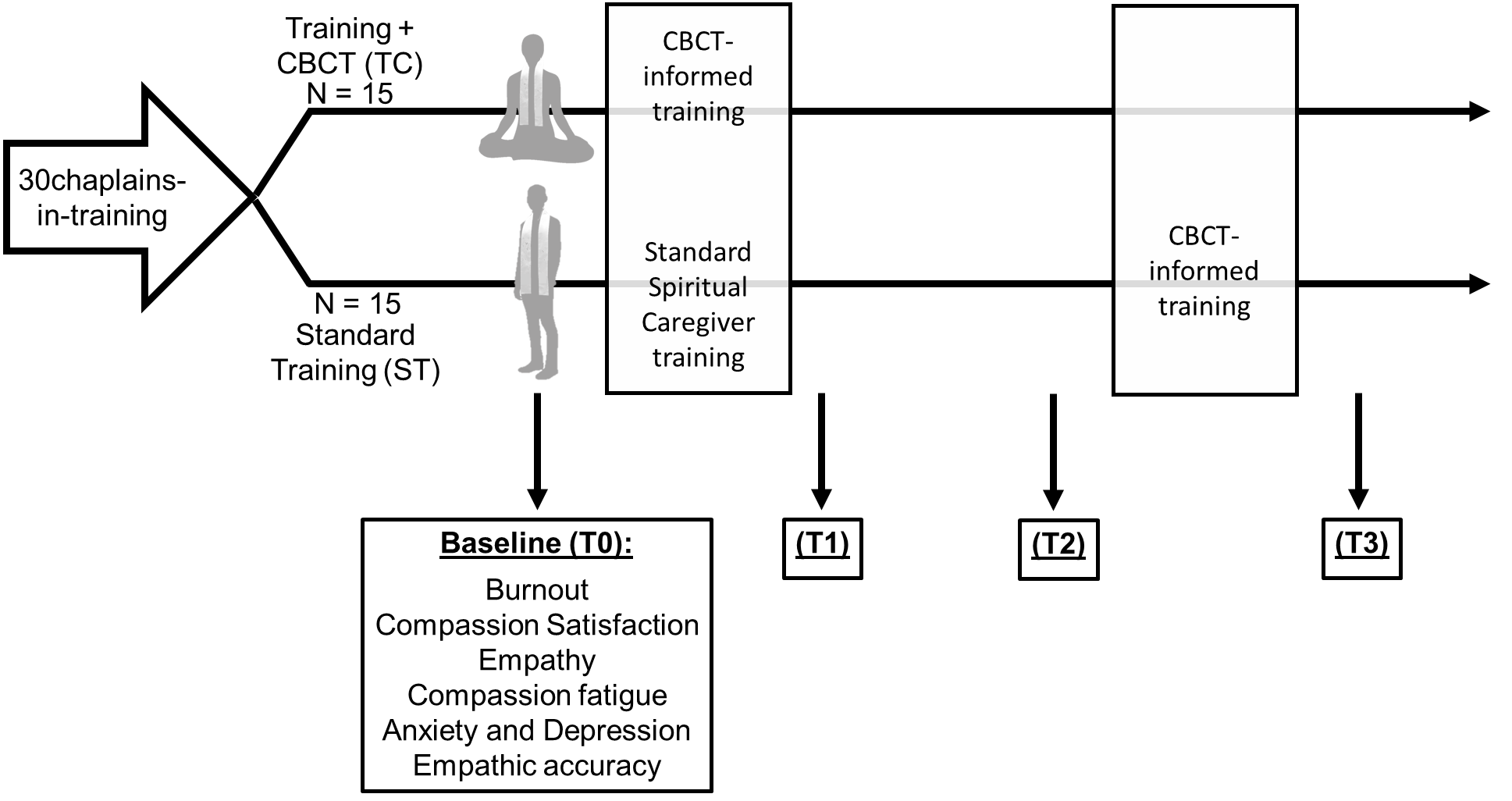


During their training program, chaplain residents will provide spiritual consults to patients throughout the Emory hospital system. We will examine whether patient (n = 300) outcomes are improved for those receiving chaplain spiritual care augmented by CBCT, in comparison to those receiving spiritual care from chaplains who receive CBCT at the end of their residency program. To this end, study personnel will shadow chaplain residents in both groups and will administer self-report assessments of negative symptomology (depression, anxiety, and loneliness) and well-being (quality of life, hope) to consented patients. In addition, study personnel will audio record chaplain-patient interactions. Patient outcomes will be gathered from retrospective chart reviews and will include use of opioid pain medication, duration of stay, patient’s denial of care, and patients’ Press Ganey satisfaction scores.

In a final step, using patient chart reviews we will examine chaplain and patient factors that may mediate the link between spiritual consults and improved patient outcomes. Specifically, we will examine whether chaplain empathic accuracy or self-reported compassion mediate the impact of spiritual care consults on patient health outcomes. We will also examine whether patients’ well-being (hope, quality of life), or negative symptoms (depression, anxiety, loneliness) mediate the impact of spiritual care consults on patient health outcomes.

We will also use the ambulatory ambulatory audio recordings of the chaplain-patient interactions to interrogate which aspects of the chaplain-patient interaction mediate improved patient outcomes (see Figure 4). Spiritual consult audio recordings will be coded by 2 trained, independent CBCT instructors for CBCT content. They will also be coded for linguistic and behavioral elements derived from existing models of compassionate interaction in the hospital environment[^9^](#_ENREF_9).

**Figure 3: Study schematic**

**Cognitively-Based Compassion Training (CBCT)**

The compassion meditation technique that will be incorporated into the spiritual caregiver training program has been adapted and secularized by the contemplative principal investigator for the project, Satya D. Negi, Ph.D., senior instructor in the Department of Religion and spiritual director of Drepung Loseling Monastery, Inc., in Atlanta. Although secular in presentation, the compassion meditation program is derived from Tibetan Buddhist mind-training (Tibetan *lojong*) practices. These practices derive largely from writings ascribed to the Indian Buddhist teachers Shantideva (8^th^ Century) and Atisha (11^th^ Century) (The Dalai Lama, 2001) and differ in important ways from the mindfulness-based practices that have received most of the scientific attention in recent years. Whereas mindfulness-based practices emphasize the development and maintenance of a non-judgmental stance toward thought processes and emotional reactions,[^10^](#_ENREF_10) lojong practices utilize a cognitive, analytic approach to challenge one’s unexamined thoughts and emotions toward other people, with the long-term goal of developing altruistic emotions and behavior towards all people [^11^](#_ENREF_11).

Lojong-based compassion meditation has two primary elements: an initial phase in which various arguments are examined that challenge one’s common sense notion of other people as falling into the categories of “friend, enemy and stranger” and a second phase in which one practices developing spontaneous feelings of empathy and love for an ever expanding circle of people, beginning with an exploration of the interdependence of one’s own life with that of others and then cultivating a sense of gratitude, even for strangers and eventually for apparent adversaries. Within the Tibetan Buddhist tradition, concentrative (i.e. shamatha) and mindfulness (i.e. vipassana) practices are typically employed as valuable preliminary techniques for establishing the focus and awareness necessary to engage in specific compassion practices [^11^](#_ENREF_11). In keeping with this tradition, in the current study subjects are introduced to attentional and mindfulness-based techniques (modules 1 - 2) to help improve attention and awareness prior to commencing specific lojong compassion practices in training modules 3-6. The training protocol is highly iterative, such that by the end of presenting the 6 modules, each student’s daily meditation practice will begin with a brief period of *shamatha* and *vipassana* to calm and focus the mind, followed by analytical practices designed to challenge unexamined assumptions regarding feelings and actions toward others with a focus on generating spontaneous empathy and compassion for themselves and others.

CBCT includes didactic teaching combined with meditations aimed at cultivating the following skills, and proceeds according to the following outline:

1. Developing Attention and Stability of Mind: The foundation for the practice is the cultivation of a basic degree of refined attention and mental stability.
2. Cultivating Insight into the Nature of Mental Experience: The stabilized mind is then employed to gain insight into the nature of the inner world of thoughts, feelings, emotions and reactions.
3. Cultivating Self-Compassion: The student participant observes the innate aspirations for happiness and wellbeing as well as those for freedom from unhappiness and dissatisfactions, i.e., which mental states contribute to fulfillment and which ones prevent it. The participant then makes a determination to emerge from the toxic mental and emotional states that promote unhappiness.

1. Developing Equanimity: Normally one tends to hold fast to categories of friends, enemies, and strangers and to react unevenly to people, based on those categories, with over-attachment, indifference and dislike. By examining these categories closely, the participant comes to understand their superficiality and learns to relate to people from a deeper perspective: everyone is alike in wanting to be happy and to avoid unhappiness.
2. Developing Appreciation and Gratitude for Others: Although people view themselves as independent, self-sufficient actors, the truth is that no one can thrive or even survive without the support of countless others. When the participant realizes interdependence with others and the many benefits which others offer every day, the participant develops appreciation and gratitude for them.
3. Developing Affection and Empathy: Deeper contemplation and insight into the ways in which myriad benefits are derived from countless others, along with awareness that this kindness should by rights be repaid, enables the participant to relate to others with a deeper sense of connectedness and affection. By relating to others with a profound sense of affection and endearment, the participant is able to empathize deeply with them. The participant cannot then bear to see others suffer any misfortune and rejoices in their happiness.

1. Realizing Wishing and Aspirational Compassion: Enhanced empathy for others, coupled with intimate awareness of their suffering and its causes, naturally gives rise to compassion: the wish for others to be free from suffering and its conditions.

1. Realizing Active Compassion for Others: In the final step, the participant is guided through a meditation designed to move from simply wishing others to be free of unhappiness to actively committing to assistance in their pursuit of happiness and freedom from suffering.
2. **Measures:**

**Chaplains**

- 1. **Demographic and attitudes toward CBCT**
- (3 min) **Interest in CBCT Survey**: will assess interest with and experience in meditation and yoga and expectancy.
- (4 min) **Demographic scale**: asks age, ethnicity, marriage status, number and ages of children, and housing status
- **Adoption:** We adapted a scale previously used[^12^](#_ENREF_12) to measure practitioner attitudes and intent to deliver an intervention. The scale includes 6 attitude items (i.e., 3 Positive Outcome and 3 Negative Process) as well as 6 items on organizational barriers to adoption of evidence-based practices. These 12 items are rated on a 5-point Likert scale from 1 (strongly disagree) to 5 (strongly agree).
- (2 min) **Attitudes about empathy scale**[**^13^**](#_ENREF_13): a 6-item likert scale questionnaire that asks people to rate their agreement with statements about the nature of empathy.
  1. **Resilience**
- (4 min) **The Depression Anxiety and Stress Scale (DASS**)[**^14^**](#_ENREF_14): is a 42-item likert-scale measure that assesses the frequency of symptoms of depression, anxiety and stress during the past week.
- (2 min) **Mental Health Continuum Short Form (MHC-SF**)[^15^](#_ENREF_15): a 14-item inventory assessing emotional well-being (<http://calmhsa.org/wp-content/uploads/2013/06/MHC-SFEnglish.pdf>)
- (4 min) **Professional Quality of Life Scale (ProQOL)**[^16^](#_ENREF_16): a 30-item inventory, the ProQOL is the most commonly used measure of the negative and positive effects of helping others who experience suffering. It has sub-scales for compassion satisfaction, burnout, and compassion fatigue.
- (2 min) **School-Burnout Inventory (SBI)**[**^17^**](#_ENREF_17)**:** a 9-item inventory that measures burnout in the context of an academic environment.
- **(6 min) Tellegen Absorption Scale (TAS)**[**^18^**](#_ENREF_18)**:** a 34-item inventory measuring openness to absorbing and self-altering experiences.
- **(3 min) Sensed Presence Questionnaire (SenPQ)**[**^19^**](#_ENREF_19)**:** a 16-item likert-scale measure assessing recent experiences of subjective feelings that an entity, individual, or being is present in the absence of clear sensory evidence. Responses to this scale has been shown to vary with meditation practice.
  1. **Compassion and Empathy**
- (15 min) **Empathic Accuracy (EA) Task**[**^8^**](#_ENREF_8)**:** a dynamic video assessment that asks subjects to continuously rate the emotions of others as they tell emotional autobiographical stories.
- (2 min) **Spiritual Meaning Scale (SMS)**[^20^](#_ENREF_20): a 15-item, likert-scale inventory that assesses the extent to which someone endorses a belief in something larger than themselves.
- (4 min) **Jefferson Scale of Empathy**[^21^](#_ENREF_21)^,^[^22^](#_ENREF_22): a 20-item scale designed to measure empathy in practicing health care professionals and health care professional students. It has been translated into 53 languages and has been used in more than 80 countries.

Total time required for completion of measures is approximately 45 minutes. All measures will be delivered digitally.

- 1. **Post Patient Consult Checklists**
- (2-4 min) **Post-Visit Checklist for CBCT:** a 41-item checklist that assesses the encounter the CBCT-trained Chaplain Resident had with the patient; including how/if the Resident prepared for the consult, assessed the patient’s state of mind, used specific tools and skills during the consult, and how well the Resident thought the encounter went.
- (2 min) **Post-Visit Checklist for non-CBCT:** a 17-item checklist that assesses the encounter the non-CBCT-trained Chaplain Resident had with the patient; including how/if the Resident prepared for the consult, assessed the patient’s state of mind, used specific tools and skills during the consult, and how well the Resident thought the encounter went.

**Patients**

- (<1 min) **NCNN Distress Thermometer**[**^23^**](#_ENREF_23)**:** a single-item distress thermometer that compares favorably with longer measures used to screen for distress.
- (1 min) **PROMIS Self-Efficacy for Managing Emotions**[**^24^**](#_ENREF_24)**:** a likert-27-item scale that measures a subject’s level of confidence to manage and control symptoms of anxiety, depression, helplessness, discouragement, frustration, disappointment, and anger.
- (2 min) **PROMIS Positive Affect**[**^25^**](#_ENREF_25)**:** a 15-item likert-scale that asseses momentary positive or rewarding affective experiences such as feelings and mood associated with pleasure, joy, elation, contentment, pride, affection, happiness, engagement and excitement.
- (4 min) **Hospital Anxiety and Depression Scale (HADS)**[**^26^**](#_ENREF_26)**:** a 14-item questionnaire that is widely used for detecting anxiety and depressive disorders.
- (2 min) **PROMIS Emotional Support**[**^27^**](#_ENREF_27)**:** a 4-item likert-scale that assessed perceived feelings of being cared for and valued as a person.
- (2 min) **PROMIS Informational Support**[**^28^**](#_ENREF_28)**:** a 4-item likert-scale that assesses perceived availability of helpful information or advice.
- (2 min) **PROMIS Social Isolation**[**^29^**](#_ENREF_29)**:** a 4-item likert-scale that assesses perceptions of being avoided, excluded, detached, disconnected from, or unknown by, others.
- (4 min) **Scottish Patient Reported Outcome Measure (PROM)**[**^30^**](#_ENREF_30)**:** an 18-item likert-scale that assesses how a hospitalized patient felt about the Pastoral Care visit both during the consult and after it. It also assesses the patients present state regarding their situation and whether they see themselves as a spiritual or religious person.

For the post-CBCT assessments (T1 for the early-start group, and T3 for the delayed-start group), in addition to the questionnaires listed above we will also ask participants to complete a questionnaire regarding their experiences with CBCT. This questionnaire will take approximately 2-4 minutes to complete. A subset of the chaplain residents will be interviewed at three time points relative to their training in CBCT. The interviews consist of open-ended questions to further query their learning experiences with CBCT. We will also collect anonymized curriculum materials completed by chaplain residents enrolled in the study. Curriculum material will be coded with a subject ID and will not be linked to personal identifiers when researchers are analyzing the data.

**Audio Recordings:**

Chaplain residents will wear a recording device that will record chaplain-patient interactions. Patients will be informed that they can stop the recording at any time and that they can ask for their recording to be deleted at any time if they no longer wish for it to be a part of the study. Audio recordings will be de-identified, such that patient and chaplain are only linked with participant IDs and identity will not be available to anyone coding the recordings. Patients and chaplain participants will complete an assent form to indicate how their recordings can be used, including whether recordings can be used for other research projects by our team, by other researchers, and for scientific presentation of these data.

1. **Data analysis**

We will analyze the data to test the hypothesis that CBCT improves chaplain residents’ empathic accuracy and resilience in a hospital setting. SAS 9.4 and SPSS 23 will be used to conduct statistical analysis of the collected data. Descriptive statistics will be used to describe selected characteristics of participants by group, expressed as frequencies and percentages. The primary outcomes of empathic accuracy, compassion fatigue, compassion satisfaction, empathy, anxiety, and depressive mood will be measured as pre-to-post differences in the measurement scores, i.e., T1 – T0. For the early-start group and T3 – T2 for the delayed-start group. We will examine the effect of CBCT by analyzing the correlated, repeated-measure data (T1 – T0) – (T3 – T2) using mixed linear models, implemented in SAS Proc Mixed. Mean pre-to-post differences will be compared across groups.

To evaluate whether the effects of CBCT persist over time, differences between mean scores on measures of empathic accuracy and resilience for the early-care group will be compared across four time points: from baseline measurement to early-group post-CBCT measurement (T1 – T0), from early-group post-CBCT measurement to delayed-group pre-CBCT measurement (T2 – T1)_,_ and from delayed-group pre-CBCT measurement to delayed-group post-CBCT measurement (T3 - T2). To evaluate whether the effects of CBCT depend on the timing of program delivery, the differences between mean scores on empathic accuracy and resilience will be compared for each group during the period following receipt of the CBCT program; that is, (T1 – T0) – (T3 – T2).

To evaluate whether CBCT-informed interventions improve patient outcomes, descriptive statistics (means, standard deviation, standard error) will be used to characterize baseline demographics and responses to self-report assessments. All data will be evaluated for normality, and appropriate procedures will be performed in cases where normality is violated. To evaluate whether CBCT-informed spiritual consults impact patients’ negative symptoms, well-being, and patient health outcomes (duration of stay and patients’ Press Ganey satisfaction scores) we will conduct independent samples t-tests. Dichotomous variables (denial of treatment, opioid use) will be evaluated using chi square tests.

To explore chaplain and patient mediators of the impact of spiritual care consults on patient outcomes, chaplain resident, patient, and chaplain-patient interactions will be tested in mediation analyses using linear regression analysis in order to examine which factors account for improvement in patient outcomes (opioid use, duration of stay, denial of care, patient satisfaction). Any chaplain variables (empathic accuracy or self-reported compassion) that are impacted by CBCT, or patient variables (negative symptoms, well-being) that are impacted by CBCT-informed modules will be entered as independent variables into linear regression analysis with group assignment, and with patient health outcomes as the dependent variable in order to examine whether chaplain, patient, or chaplain-patient interaction variables render group assignment insignificant in accounting for variance in patient health outcomes[^31^](#_ENREF_31).

1. **Compensation:**

No compensation will be provided to participants.

1. **Risks to participation:**

Chaplain residents and Emory Healthcare patients may experience some emotional discomfort while completing the questionnaires. To minimize this risk, all participants will be informed that they are free to skip any questions that are upsetting and that they can stop the questions at any time. In addition, they will be provided help in contacting the Emory Faculty and Staff Assistance Program (FSAP).

In addition, the questionnaires that subjects will fill out as part of this study will contain personal information, and participants may have private pieces of audio recorded.

We acknowledge that it could be embarrassing if this information were to become public or fall into the hands of someone not associated with the study. We will take steps to prevent this from happening. All self-report data will be de-identified and stored by a study number, such that these data will be anonymous at all times during data entry and analysis. In addition, all data, including personal identifiers (i.e. name, address, email contact details), will be maintained in secure password-protected electronic files stored on a secure computer system that can only be accessed by study researchers.

1. **Procedures for minimizing risks:**

To minimize the risk that participants become upset while completing questionnaires, the residents will be informed that they are free to skip any questions that are upsetting and that they can stop the questions at any time. In addition, they will be provided help in contacting the Emory Faculty and Staff Assistance Program (FSAP).

Personal identifiers (i.e. name, address, email contact details) will be maintained in secure password-protected electronic files. All of the data collected for these studies will be kept strictly confidential. Under no circumstances will individually identifiable data be released to anyone without the written consent of the subject. Emory Healthcare administrators will not have access to questionnaire data. Results will be discussed with the subjects at their request.

1. **Risk-Benefit Ratio:**

Other people may potentially benefit from what this study reveals. It is hoped that such knowledge will ultimately lead to a better understanding of how CBCT affects the spiritual caregiver in a hospital environment and may inform the application of CBCT to improve patient care. These potential benefits outweigh the minimal risk this research poses to subjects.

1. **Data Safety Monitoring Plan:**

Human subjects’ data to be collected includes responses to questionnaires. Subject data will be identified by ID number, with only the PI and IRB-approved personnel working in her lab having access to the list linking subject names and ID #s. This list will be saved as a password-protected computer file. All data will be collected specifically for the purpose of this research project.

All data will be stored on a secure computer system that can only be accessed by study researchers. Questionnaires will be stored by study number and not by subject name. Any paper data will be stored in a locked filing cabinet, to which only investigators in this study will have access. All electronic data will be stored in a password-protected Emory database, which only Dr. Mascaro and the member(s) of the research team assisting with data analysis have access to. The database is monitored by Emory computing services, and any problems or breaches will be directed to Dr. Mascaro’s attention.

All AEs that occur during the course of the study, either directly related to the CBCT program or unrelated, will be reported to the Emory IRB. Any SAEs will be reported within 24 hours according to standard regulations, and will be reported to funding agencies.

1. **Benefits to subject or future benefits:**

Chaplain residents participating in the study may benefit from the introspection and learning associated with completing the study questionnaires. Chaplain resident participants will also likely benefit from exposure to research methodology as part of their CPE experience.

Other people may potentially benefit from what this study reveals. It is hoped that the knowledge gained will ultimately lead to a better understanding of how CBCT affects the spiritual caregiver in a hospital environment and inform the application of CBCT to improve patient care.

1. **Training:**

All Study personnel are CITI certified and will maintain CITI certification throughout their involvement in the study. Data collection and statistical analyses will be carried out by Dr. Mascaro or her research assistants.

1. **Confidentiality:**

The study will keep any records that are produced private. Study number will be used rather than subject’s name on study records where applicable. Subject’s name and other facts that might point to subject will not appear when this study is presented or results are reported.

1. **Informed consent:**

Written informed consent will be obtained at the Emory Healthcare campuses by Dr. Mascaro or approved study personnel prior to showing the empathic accuracy videos or administering questionnaires.

1. **Plans to inform participants of new findings or research results that might affect health:**

If researchers learn any new information that might affect the risk/benefit ratio for study participation, research subjects will be notified immediately—either in person for subjects still in protocol or by phone and/or email for subjects who have completed or dropped out of the protocol.

**References:**

1. Mascaro JS, Rilling JK, Tenzin Negi L, Raison CL. Compassion meditation enhances empathic accuracy and related neural activity. *Social Cognitive and Affective Neuroscience.* 2013;8(1):48-55.

2. Mascaro JS, Kelley S, Darcher A, et al. Meditation buffers medical student compassion from the deleterious effects of depression. *The Journal of Positive Psychology.* 2016:1-10.

3. Desbordes G, Negi LT, Pace TWW, Wallace AB, Raison C, Schwartz EL. Effects of mindful-attention and compassion meditation training on amygdala response to emotional stimuli in an ordinary, non-meditative state. *Front Hum Neurosci.* 2012;6(292):1-15.

4. Pace TWW, Negi LT, Adame DD, et al. Effect of compassion meditation on neuroendocrine, innate immune and behavioral responses to psychosocial stress. *Psychoneuroendocrinology.* 2009;34(1):87-98.

5. Pace TWW, Negi LT, Dodson-Lavelle B, et al. Engagement with Cognitively-Based Compassion Training is associated with reduced salivary C-reactive protein from before to after training in foster care program adolescents. *Psychoneuroendocrinology.* 2012(0).

6. Galek K, Flannelly KJ, Greene PB, Kudler T. Burnout, secondary traumatic stress, and social support. *Pastoral Psychology.* 2011;60(5):633-649.

7. Mascaro JS, Darcher A, Negi LT, Raison CL. The neural mediators of kindness-based meditation: a theoretical model. *Frontiers in psychology.* 2015;6(109).

8. Zaki J, Weber J, Bolger N, Ochsner K. The neural bases of empathic accuracy. *Proceedings of the National Academy of Sciences.* 2009;106(27):11382-11387.

9. Sinclair S, McClement S, Raffin-Bouchal S, et al. Compassion in health care: An empirical model. *Journal of pain and symptom management.* 2016;51(2):193-203.

10. Kabat-Zinn J. *Full catastrophe living: using the wisdom of your body and mind to face stress, pain, and illness.* New York: Dell Publishing; 1991.

11. The Dalai Lama HH. *An Open Heart: Practicing Compassion in Everyday Life.* New York: Little, Brown and Company; 2001.

12. Haug NA, Shopshire M, Tajima B, Gruber V, Guydish J. Adoption of evidence-based practices among substance abuse treatment providers. *Journal of Drug Education.* 2008;38(2):181-192.

13. Schumann K, Zaki J, Dweck CS. Addressing the empathy deficit: Beliefs about the malleability of empathy predict effortful responses when empathy is challenging. *Journal of Personality and Social Psychology.* 2014;107(3):475.

14. Lovibond PF, Lovibond SH. The Structure of Negative Emotional States - Comparison of the Depression Anxiety Stress Scales (DASS) with the Beck Depression and Anxiety Inventories. *Behav Res Ther.* 1995;33(3):335-343.

15. Lamers S, Westerhof GJ, Bohlmeijer ET, ten Klooster PM, Keyes CL. Evaluating the psychometric properties of the mental health Continuum‐Short Form (MHC‐SF). *J Clin Psychol.* 2011;67(1):99-110.

16. Stamm BH. The ProQOL manual. *Retrieved July.* 2005;16:2007.

17. Salmela-Aro K, Kiuru N, Leskinen E, Nurmi J-E. School burnout inventory (SBI) reliability and validity. *European Journal of Psychological Assessment.* 2009;25(1):48-57.

18. Tellegen A, Atkinson G. Openness to absorbing and self-altering experiences (" absorption"), a trait related to hypnotic susceptibility. *Journal of abnormal psychology.* 1974;83(3):268.

19. Barnby JM, Bell V. The Sensed Presence Questionnaire (SenPQ): initial psychometric validation of a measure of the “Sensed Presence” experience. *PeerJ.* 2017;5:e3149.

20. Mascaro N, Rosen DH, Morey LC. The development, construct validity, and clinical utility of the spiritual meaning scale. *Personality and Individual Differences.* 2004;37(4):845-860.

21. Hojat M, Vergare M, Isenberg G, Cohen M, Spandorfer J. Underlying construct of empathy, optimism, and burnout in medical students. *International Journal of Medical Education.* 2015;6:12-16.

22. Hojat M, Vergare MJ, Maxwell K, et al. The devil is in the third year: a longitudinal study of erosion of empathy in medical school. *Academic Medicine.* 2009;84(9):1182-1191.

23. Jacobsen PB, Donovan KA, Trask PC, et al. Screening for psychologic distress in ambulatory cancer patients. *Cancer.* 2005;103(7):1494-1502.

24. Gruber-Baldini AL, Velozo C, Romero S, Shulman LM. Validation of the PROMIS((R)) measures of self-efficacy for managing chronic conditions. *Qual Life Res.* 2017;26(7):1915-1924.

25. A Brief Guide to the PROMIS Positive Affect Instruments. 2018.

26. Snaith RP. The Hospital Anxiety And Depression Scale. *Health Qual Life Outcomes.* 2003;1:29.

27. A Brief Guide to the PROMIS Emotional Support Instruments. 2015.

28. A Brief Guide to the PROMIS Informational Support v2.0 Instruments. 2015.

29. A Brief Guide to the PROMIS Social Isolation Instruments. 2015.

30. Snowden A, Telfer I. Patient Reported Outcome Measure of Spiritual Care as Delivered by Chaplains. *J Health Care Chaplaincy.* 2017;23(4):131-155.

31. Baron RM, Kenny DA. The moderator–mediator variable distinction in social psychological research: Conceptual, strategic, and statistical considerations. *Journal of Personality and Social Psychology.* 1986;51(6):1173-1182.
